# Catch me if you can: capturing microbial community transformation by extracellular DNA using Hi-C sequencing

**DOI:** 10.1007/s10482-023-01834-z

**Published:** 2023-05-08

**Authors:** David Calderón-Franco, Mark C. M. van Loosdrecht, Thomas Abeel, David G. Weissbrodt

**Affiliations:** 1 Department of Biotechnology, Delft University of Technology, Delft, The Netherlands; 2 Delft Bioinformatics Lab, Delft University of Technology, Delft, The Netherlands; 3 Infectious Disease and Microbiome Program, Broad Institute of MIT and Harvard, Cambridge, MA USA; 4 Department of Biotechnology and Food Science, Norwegian University of Science and Technology, Trondheim, Norway

**Keywords:** Hi-C sequencing, Mixed cultures, Antibiotic resistance, Transformation, Plasmids

## Abstract

The transformation of environmental microorganisms by extracellular DNA is an overlooked mechanism of horizontal gene transfer and evolution. It initiates the acquisition of exogenous genes and propagates antimicrobial resistance alongside vertical and conjugative transfers. We combined mixed-culture biotechnology and Hi-C sequencing to elucidate the transformation of wastewater microorganisms with a synthetic plasmid encoding GFP and kanamycin resistance genes, in the mixed culture of chemostats exposed to kanamycin at concentrations representing wastewater, gut and polluted environments (0.01–2.5–50–100 mg L^−1^). We found that the phylogenetically distant Gram-negative *Runella* (102 Hi-C links), *Bosea* (35), *Gemmobacter* (33) and *Zoogloea* (24) spp., and Gram-positive *Microbacterium* sp. (90) were transformed by the foreign plasmid, under high antibiotic exposure (50 mg L^−1^). In addition, the antibiotic pressure shifted the origin of aminoglycoside resistance genes from genomic DNA to mobile genetic elements on plasmids accumulating in microorganisms. These results reveal the power of Hi-C sequencing to catch and surveil the transfer of xenogenetic elements inside microbiomes.

**Supplementary Information:**

The online version contains supplementary material available at 10.1007/s10482-023-01834-z.

## Introduction

Antimicrobial resistance (AMR) is one major public health threats of the twenty-first century. By 2050, ten million lives will be at risk per year due to the rise of infections by antibiotic resistance bacteria (ARB) if no mitigation efforts are engaged (O’Neill 2016). In the One Health context, wastewater treatment plants (WWTPs) face the cocktail of chemical, pharmaceutical and biological agents that are transported by used waters discharged from households, healthcare, industrial and agricultural settings, and that eventually meet in a complex microbial sludge.

Microorganisms in natural and man-made systems constantly evolve under environmental stressors by horizontal gene transfer (HGT) using conjugation, transduction, endocytosis or transformation processes (Aminov 2011; Kapteijn et al. 2022; Li and Zhang 2022). Microbes benefit from transferring genetic information that code for AMR via mobile genetic elements (MGEs) like plasmids, integrons, transposons, and conjugative and integrative elements (Van Dijk et al. 2020). The transformation of microorganisms by extracellular free DNA (exDNA) materials in microbial communities remains a definite challenge. Environmental conditions that trigger microbial competence (*e.g.*, seasonal changes, nutrient limitations, antibiotic concentrations) remain vastly unclear, together with the microbial hosts and vectors of MGEs that lead to AMR propagation (Moralez et al. 2021).

Recently, we highlighted that exDNA in wastewater is a rich pool of MGEs (65%; 5–9 µg exDNA L^−1^) (Calderón-Franco et al. 2021) that can co-localize ARGs (Calderón-Franco et al. 2022). Detecting the uptake of exDNA materials from the environment by natural transformation is an important motivation (Ikuma and Rehmann 2020). Such cell-free DNAs can disseminate AMR determinants from wastewater environments into aquatic reservoirs and drinking water resources (Woegerbauer et al. 2020).

Natural transformation is the process by which bacteria actively take up and integrate exDNA, providing a source of genetic diversity (Lin et al. 2009). Naturally competent bacteria actively pull DNA fragments from their environment into their cells (Mell and Redfield 2014). Cells take up exDNA according to their nutritional needs, genomic DNA damage, recombination ability in the chromosomal DNA, and the recombination effects on their microbial fitness, like beneficial physiological traits acquired through antibiotic resistance genes (ARGs). (Pietramellara et al. 2009; Overballe-Petersen et al. 2013; Winter et al. 2021). Experimental demonstrations of natural competence have been limited to only a few dozen species scattered across the bacterial tree of life and examined in pure cultures (Lorenz and Wackernagel 1994; Håvarstein 1998). These genetic transformation assays are sensitive, but can only be conducted with cultivable species, and that harbour a selectable genetic marker like an ARG. These cultivation-dependent methods cannot detect competence in complex microbial communities, where DNA uptake rarely leads to recombination or episomal integration. Most studies further tried to quantify HGT via conjugation (Pérez-Mendoza and de la Cruz 2009; Lopatkin et al. 2016a; Pallares-Vega et al. 2021) or transformation (Nordgård et al. 2007; Cooper et al. 2017; Domenech et al. 2020) using controlled synthetic consortia and biofilms of engineered or well-characterized strains. However, these methods cannot uncover in-situ natural phenomena of HGT inside microbial communities. Conditions that induce transformation have equally not been unravelled yet.

Environmental samples such as activated sludge are ideal to study the natural competence potential of bacteria among their microbial diversity. Activated sludge can be composed of several potential hosts that encompass multiple mechanisms to maintain and transfer plasmids. Once an organism is transformed with the exogenous synthetic plasmid, it can disseminate it by vertical gene transfer or by conjugation to other populations if mobilizable (*mob*) and transfer (*tra*) operons are present on the plasmid or integrated in the genome (Simon et al. 1983; Bottery 2022).

The investigation of HGT and natural transformation in complex microbial ecosystems requires advanced sequencing technologies to identify microorganisms that take up, carry, and transfer specific MGEs (Moralez et al. 2021). Hi-C sequencing provides this opportunity by proximity ligation of MGEs with the chromosomal DNA of microorganisms prior to extraction and metagenomic sequencing of the ligated DNA pool. Resistomes, plasmidomes and viromes have been linked to the microbiomes of wastewater (Stalder et al. 2019), rumen (Bickhart et al. 2019) and stem cell transplantation patients (Kent et al. 2020), highlighting the diversity of microorganisms potentially able to transfer resistance determinant fragments.

Aminoglycoside antibiotics promote microbial competence by inducing decoding errors during the translation of tRNA into amino acids. Kanamycin inhibits protein synthesis by binding to the A site of the 16S rRNA in the 30S ribosomal subunit and generates misfolded proteins that activate the high temperature requirement A serine protease (HtrA). This triggers a cascade of reactions involving interactions between competence-stimulating proteins (e.g., ComC, ComAB, CSP) that eventually launches the competence state (Stevens et al. 2011; Slager et al. 2014).

In this work, we show that wastewater microorganisms can get naturally transformed with an exogenous synthetic rolling-circle plasmid encoding green-fluorescence protein (GFP) and kanamycin resistance (*kanR*) genes and deprived of any conjugative property. This was demonstrated in continuous mixed cultures inoculated with activated sludge and exposed to increasing concentrations of kanamycin (0.01–2.5–50–100 mg L^−1^) representing antibiotic levels in water, wastewater, gut, and polluted environments. Furthermore, we identified that the microorganisms selected under high antibiotic pressure mobilised natural plasmids that carry aminoglycoside resistance genes, enabling them to survive and grow in such heavily impacted environment.

## Methods

### Mixed-culture bioreactor systems and operation

#### Chemostats

**Fig. 1:**
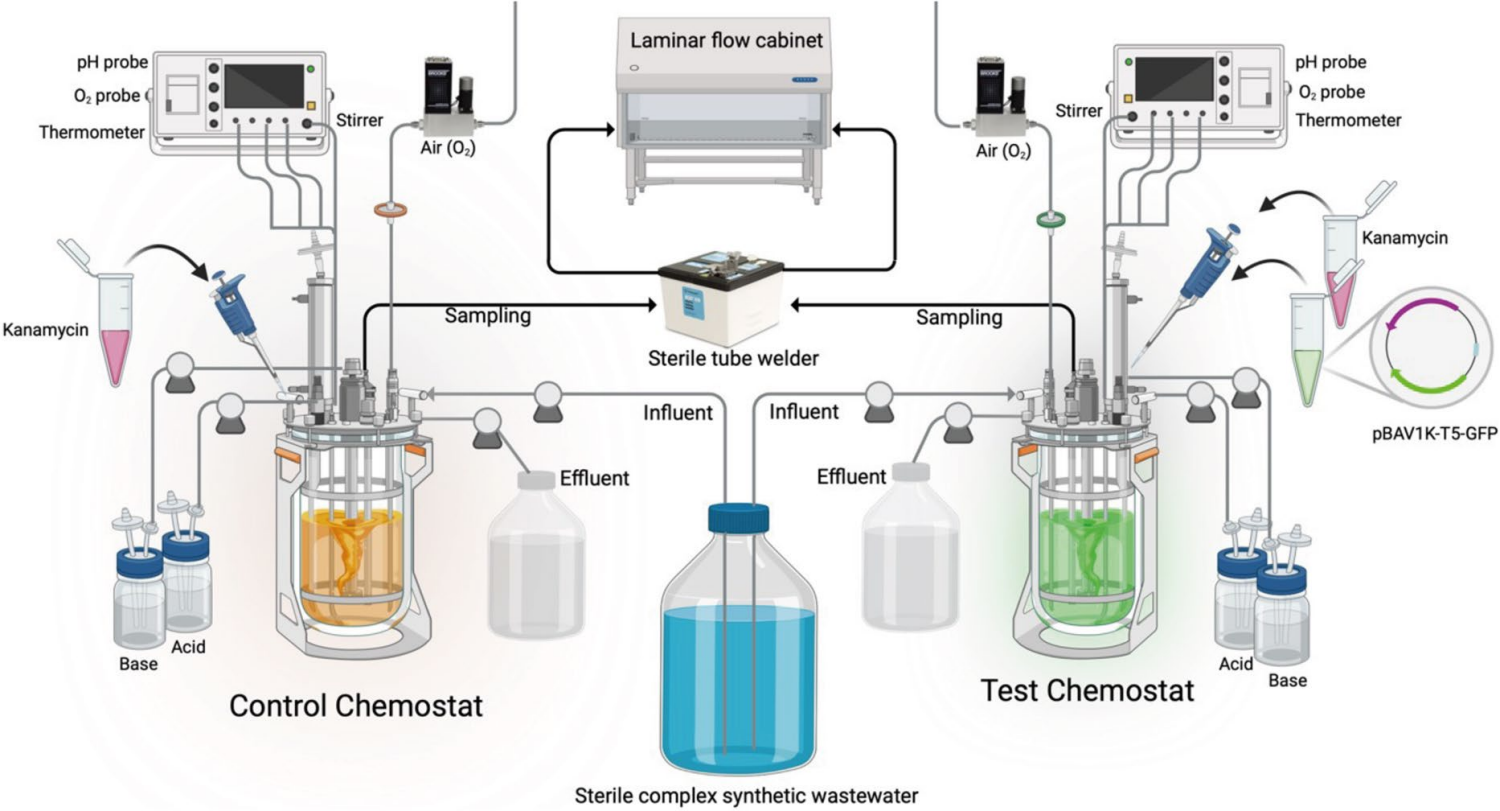
Schematic representation of the set containing two continuously stirred bioreactors (chemostats). The control chemostat was spike with only kanamycin. In the test chemostat, kanamycin and the synthetic plasmid (pBAV1K-T5-GFP) were spiked daily. This experimental setting was embedded in a biosafety level II laboratory, where samples had to be extracted via a sterile tube welder and handled under sterility in a laminar flow cabinet

A control chemostat and a test chemostat (both of 1 L total volume and 0.7 L working volume) were operated identically in parallel, under axenic conditions (close environment), aerobically, at room temperature (23 ± 2 °C), fed with a complex synthetic wastewater (Supplementary material) at a hydraulic retention time (HRT) of 1 day (*i.e.*, flowrate of 1 L d^−1^ or 0.695 mL min^−1^, and dilution rate of 1 d^−1^ or 0.0417 h^−1^), and mixed at 600 rpm by mechanical stirring. The dissolved oxygen concentration was controlled with a mass flow controller (Brooks, USA), delivering a flowrate of 0.7 L air min^−1^. Both chemostats were equipped with oxygen sensors (AppliSens, Poland), thermometers, and pH probes (Mettler Toledo, USA). pH was maintained at 7.0 ± 0.5 by addition of HCl or NaOH at 1 mol L^−1^ each. All samples were taken in sterility using a tube welder (Tekyard, USA). The off-gas from the reactor was filtered-sterilized before release. The effluent was collected in a closed vessel and always autoclaved before discarding. A schematic representation of the equipment is provided in Fig. 1.

#### Inoculum

The reactors were inoculated at a low initial concentration of biomass of 0.043 g VSS L^−1^, by seeding 10 mL of activated sludge (3 g VSS L^−1^) collected from WWTP Amsterdam-West (The Netherlands), which is operated for complete biological nutrient removal. The inoculated biomass was equilibrated to the chemostat operation in both reactors over 5 HRTs in the absence of antibiotics.

#### Antibiotic supply

After acclimation, the reactors were run for 46 days under increasing concentrations of the model antibiotic kanamycin (Thermo Fisher Scientific, USA) from 0.01 to 2.5, 50, and 100 mg L^−1^. Kanamycin stocks were done in distilled water. Antibiotic loadings were changed after maintaining each condition over 8–10 HRTs. Kanamycin was spiked daily in both chemostats simultaneously (Figure S1).

#### Plasmid spikes

In the test reactor only, the rolling circle plasmid pBAV1K-T5-GFP containing genes coding for resistance to kanamycin and a green-fluorescent protein (GFP) as a reporter gene (Bryksin and Matsumura 2010) was spiked daily (5 µg L^−1^) for 37 days. The Monarch Plasmid Miniprep Kit (New England BioLabs, USA) was used to isolate plasmid DNA. This plasmid backbone has already been used for natural transformation in inter-species studies (Cooper et al. 2017) and cannot be mobilized as it does not encode mobility nor mating pair formation genes that would allow the plasmid, once transformed, to conjugate. This plasmid has been proven to positively replicate in *Acinetobacter baumannii, Streptococcus pyogenes and Francisella novicida*(Bryksin and Matsumura 2010). The plasmid was not spiked over the additional last 10 days of the experiment, to assess if the plasmid would remain accumulated in the mixed culture or washed out due to the continuous operation. The spikes of antibiotics and plasmid were injected through sterile luer connections.

#### Complex synthetic wastewater composition

A complex synthetic wastewater of a controlled composition mimicking real wastewater was prepared according to a previous work (Layer et al. 2019) that has also been applied in conjugative experiments (Pallares-Vega et al. 2021). The detailed influent composition is shown in Tables S1 and S2 in the Supplementary Material. Briefly, this medium was composed of 1/3 volatile fatty acids (1/6 acetate + 1/6 propionate), 1/3 soluble and fermentable substrates (1/6 glucose, 1/6 amino acids), and 2 particulate substrates (1/6 peptone, 1/6 starch) in equal equivalents of chemical oxygen demand (COD). Amino acids were composed of L-alanine, L-arginine, L-aspartic acid, L-glutamic acid, L-leucine, L-proline, and glycine in COD equivalents. Particulate substrates were peptone from casein, digested with trypsin (Carl Roth, Germany), and starch made from wheat (Merck Sigma, Germany). Nitrogen was supplied as a combination of soluble ammonium chloride and nitrogen from the aforementioned amino acids and peptone. Phosphorus was composed of soluble orthophosphate.

### Analytical methods for the measurements of substrates and biomass concentrations

#### Chemical analyses of the liquid phase

The mixed liquor of the chemostats was sampled as volumes of 11 mL from the effluent in triplicates at a sample frequency every 2 days. 1 mL was centrifuged at 6000×*g* 1 min and supernatants filtered through 0.2 µm PVDF membrane filters (Pall, USA), and the filtrates were used for chemical analyses of the liquid phase. Acetate, propionate, and glucose concentrations were measured using a high-performance liquid chromatograph (HPLC; Vanquish™ System, Thermo Fisher Scientific, USA) using an Aminex HPX-87H column (BioRad, USA) maintained at 59 °C and coupled to an ultraviolet detector at 210 nm (Waters, USA) and a refractive index detector (Waters, USA). A solution of phosphoric acid at 1.5 mmol L^–1^ was used as eluent. Total nitrogen (5–40 mg L^−1^ TN range) and phosphate (0.5–5.0 mg L^−1^ PO_4_^3−^-P) were measured with colorimetric-spectrophotometric cuvette tests (Hach-Lange, USA).

#### Biomass analyses

The concentrations of total suspended solids (TSS) and volatile suspended solids (VSS) of the mixed liquors were analyzed according to Standard Methods (APHA 2005). The other 10 mL obtained from the mixed liquor every two days for chemical analyses were used for TSS and VSS measurements.

### Preliminary control of plasmid transformation in pure cultures

*Escherichia coli* K12 and *Bacillus subtilis* str. 168 were used as preliminary controls to verify that the plasmid could transform and express in Gram-negative and Gram-positive microorganisms. *E. coli* cells were electroporated, and *B. subtilis* was transformed with a starvation-induced method (Bennallack et al. 2014) with the plasmid and plated in Luria–Bertani (LB) medium with kanamycin (50 mg L^−1^) to select for positive transformants. Details on electroporation and starvation-induced transformation can be found in Supplementary Material. Microscopic bright field and fluorescent pictures of positively transformed *E. coli* and *B. subtilis* can be found in Figure S2.

### Quantitative PCR analysis of kanamycin resistance and GFP marker genes

All qPCR reactions were conducted in 20 µL, including IQ™ SYBR green supermix BioRad 1x. The sets of forward and reverse primers used to amplify the green fluorescent protein (GFP) gene and the kanamycin resistance (*kanR*) gene were retrieved from (Bryksin and Matsumura 2010) and summarized in Tables 1 and S3. A volume of 2 µL of DNA template was added to each reaction, and the reaction volume was completed to 20 µL with DNase/RNase free Water (Sigma Aldrich, UK). All reactions were performed in a qTOWER3 Real-time PCR machine (Westburg, DE) according to the following PCR cycles: 95 °C for 5 min followed by 40 cycles at 95 °C for 15 s and 60 °C for 30 s.

**Table 1:** Primers used for qPCR analyses

Gene	Primer forward (5′ → 3′)	Primer reverse (5′ → 3′)
16S rRNA	ACTCCTACGGGAGGCAGCAG	ATTACCGCGGCTGCTGG
GFP	TTCAATGCTTTTCCCGTTATCC	CGTCTTGTAGTTCCCGTCATC
*kanR*	CACTTACTTTGCCATCTTTCAC	CGCTTAGCCGAATTGGATTAC

To check the specificity of the reaction, a melting curve was performed from 65 to 95 °C at a temperature gradient of + 0.5 °C (5 s)^−1^. Synthetic DNA fragments in the form of BioBricks (IDT, USA) containing each target gene were used as a positive control to create the standard curves. Serial dilutions of gene fragments were performed in sheared salmon sperm DNA at 5 µg mL^−1^ (m/v) (Thermofisher, LT) diluted in Tris–EDTA (TE) buffer at pH 8.0. Every sample was analyzed in technical triplicates. Standard curves were included in each PCR plate with at least six serial dilution points and technical duplicates. An average standard curve based on a standard curve from every run was created for every gene set. Gene concentration values were then calculated from the standard curve mentioned above.

### Analysis of microbial populations dynamics by 16S rRNA gene amplicon sequencing

Changes in compositions of the microbial communities of the two chemostats during antibiotic regime shifts were analyzed by amplicon sequencing. Volumes of 5 mL of mixed liquors were taken at the end of each antibiotic concentration period (*i.e.*, at steady states) in 15 mL Falcon tubes. Cell pellets were obtained by centrifugation at 6000×*g* during 1 min. DNA was extracted from the samples’ cell pellets using the PowerSoil microbial extraction kit (Qiagen Inc., Germany), following manufacturer’s instructions. The DNA content of the extracts was quantified using a Qubit 4 (Thermo Fisher Scientific, United States). The DNA extracts were preserved at − 20 °C pending amplicon sequencing analyses.

The DNA extracts were sent to Novogene Ldt (Novogene, Hong Kong) for the V3-V4 16SrRNA gene hypervariable regions (position 341F (CCTAYGGGRBGCASCAG) -806R (GGACTACNNGGGTATCTAAT)) on a MiSeq desktop sequencing platform (Illumina, San Diego, USA). The raw sequencing reads were processed by Novogene Ltd and quality filtered using the QIIME software (Caporaso et al. 2010). Chimeric sequences were removed using UCHIME (Edgar et al. 2011), and sequences with ≥ 97% identity were assigned to the same operational taxonomic units (OTUs) using UPARSE (Edgar 2013). Each OTU was taxonomically annotated using the Mothur software against the SSU rRNA database of the SILVA Database (Quast et al. 2013). The heatmap of relative abundances was generated using the R package “ampvis2” v2.7.31 (Andersen et al. 2018).

### Microbiome profiling by metagenomics

The same biomass samples selected for Hi-C metagenomics sequencing (collected at 2.5 and 50 mg Kan L^−1^) were sequenced in parallel by conventional metagenomics to profile their microbiomes at high resolution. Samples were submitted to and sequenced by Phase Genomics (USA). Both the shotgun library and the Hi-C library were sequenced on the same platform. The details are given in the sections hereafter. Classification with Kraken2.0 (Wood et al. 2019) was performed on pair-end mode on the quality-controlled short reads, using the Microbial Database for Activated Sludge (MiDAS) (Nierychlo et al. 2020). The taxonomic classification outcomes from Kraken2.0 were converted into a BIOM file using the kraken-biom (Dabdoub 2016) tool to explore metagenomics classification datasets via the “MicrobiotaProcess” package v1.6.6. in R (Xu et al. 2022). Relative abundances were calculated by dividing the reads mapped to a specific species by the total number of reads. Biomass was sampled from the test reactor during stationary periods at low (2.5 mg Kan L^−1^ on day 18; sample RT.D18) and high (50 mg Kan L^−1^ on day 28; sample RT.D28) antibiotic exposures for metagenomics and Hi-C sequencing.

### Hi-C sequencing

**Fig. 2:**
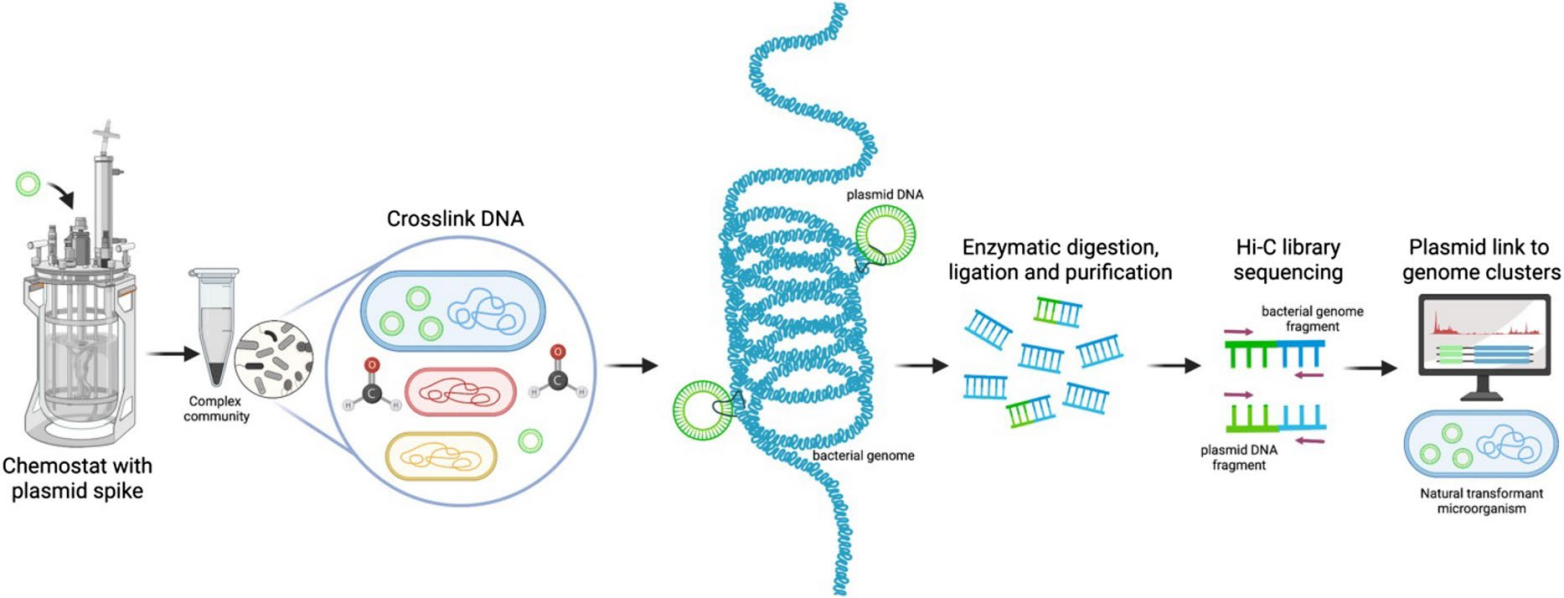
Schematic representation of the Hi-C deconvolution process. Plasmids were cross-linked to chromosomal DNA inside microorganisms of the microbial community with formaldehyde before cell lysis. DNA extract was digested enzymatically, biotinylated, ligated, and purified. To generate the Hi-C library, the resultant fraction was sequenced and used to create Hi-C links that helped deconvolute contigs into genome clusters, including chromosomes and plasmids

Hi-C libraries were prepared as explained in (Burton et al. 2014) and summarized in Fig. 2.

#### Biomass sampling

Biomass samples were collected as volumes of 10 mL of mixed liquor at the end of the antibiotic treatment periods at 2.5, and 50 mg Kan L^−1^ from the test reactor spiked with the plasmid and transferred on ice. The biomass was aliquoted in two 1.5 mL Eppendorf. One sample was used for Hi-C library generation, while the other was used for standard metagenomic library preparation.

#### Biomass sample conditioning for Hi-C analysis

A volume of 1.5 mL of the collected biomass sample was resuspended using 13.5 mL of solution of a commercial 1% formaldehyde-phosphate-buffered saline (F-PBS) (Alfa Aesar—Thermo Fischer Scientific, USA) in a 15 mL Falcon tube under biosafety level II conditions. Formaldehyde was used to generate covalent links between spatially adjacent genetic segments (Fig. 2). The resuspended sample was incubated at room temperature for 30 min with periodic mixing (every 5 min) before adding glycine at a final concentration of 1 g/100 mL to quench the reaction and further incubating the mixture at room temperature for 15 min with periodic mixing. The last step involved a series of three spin down (6000×*g*, 1 min) and rinses with PBS of the pellet. Briefly, the sample was spun down for 2 min at 6000×*g*, rinsed with PBS, and spun down again (5 min at 6000×*g*) before removing the supernatant. The final pellet was kept frozen at − 20 °C. Crosslinking is intentionally done prior to cell lysis, then crosslinking does not occur with anything located outside intact cells. Therefore, extracellular plasmids will be prevented from crosslinking to any intracellular DNA.

#### Hi-C library preparation and sequencing

Hi-C libraries were prepared with the Phase Genomics ProxiMeta Hi-C v4.0 Kit using the manufacturer-provided protocol (Lieberman-Aiden et al. 2009). Briefly, the intracellular DNA pool (comprising the genomic DNA and the accessory genome, i.e., MGEs) of the microbial cells present in each of the two selected samples were crosslinked using the formaldehyde solution. These pre-treated biomass samples were submitted to Phase Genomics for library preparation and sequencing. Cells were bead-beated to release the cross-linked DNA. The released cross-linked DNA was digested using the Sau3AI and MlucI restriction enzymes simultaneously, and proximity ligated with biotinylated nucleotides to create chimeric molecules composed of fragments from different regions of genomes and plasmids that were physically proximal in vivo. Proximity ligated DNA molecules were pulled down with streptavidin beads and processed into an Illumina-compatible sequencing library. Separately, using an aliquot of the original samples, DNA was extracted with a ZYMObiomics DNA miniprep kit (Zymo Research, USA) and a metagenomic shotgun library was prepared using ProxiMeta library preparation reagents.

Sequencing was performed on an Illumina NovaSeq generating PE150 read pairs for both the shotgun libraries and the Hi-C libraries obtained from the aliquots of each of the two biomasses collected at 2.5 and 50 mg Kan L^−1^. Hi-C and shotgun metagenomic sequencing files were uploaded to the Phase Genomics cloud-based bioinformatics platform for subsequent analysis.

### Processing of shotgun metagenomics and Hi-C metagenomics datasets

#### Quality control of sequenced reads

After sequencing, datasets containing 4 paired-end read samples with an average of 78 M reads for the Hi-C samples, and 160 M reads for the non-crosslinked samples (i.e., shotgun) were obtained. The quality of the Illumina reads was assessed using FastQC version 0.11.9 with default parameters (Andrews 2010). Shotgun reads were filtered and trimmed for quality and normalized using fastp v0.19.6 (Chen et al. 2018).

#### Assembly of shotgun sequence reads

The trimmed reads were assembled into contigs using MEGAHIT v1.2.9 (Li et al. 2015) for the resistome analysis. The trimmed reads were assembled into contigs using metaSPAdes version 3.14.1 (Nurk et al. 2017) on meta mode on default parameters for following discordant reads analysis (to quantify interactions plasmid:bacteria). We used these two assemblers to verify which one resulted in positive results for plasmid-host detection (only metaSPAdes displayed positive results).

#### Processing of the Hi-C reads

Each set of Hi-C reads was mapped to the metagenomic assemblies to generate a SAM file containing the information of the assembly and the Hi-C links. Mapping was done using the Burrows-Wheeler alignment tool BWA-MEM v0.7.17-r1188 (Li and Durbin 2009). During mapping with BWA MEM, read pairing and mate-pair rescue functions were disabled and primary alignments were forced to be aligned with the lowest read coordinate (5’ end) (options: -5SP) (Demaere and Darling 2019). SAMBLASTER v0.1.26 (Faust and Hall 2014) was used to flag PCR duplicates, which were later excluded from the analysis. Alignments were then filtered with samtools v1.13 (Li et al. 2009) using the -F 2304 filtering flag to remove non-primary and secondary alignments.

#### Deconvolution of the Hi-C data for aminoglycoside resistome analysis

Metagenome deconvolution was performed with ProxiMeta (Press et al. 2017; Stewart et al. 2018), creating putative genome and genome fragment clusters (Figs. S3–S4). Clusters (also known as metagenome-assembled genomes or MAGs) were assessed for quality using CheckM v1.1.10 (Parks et al. 2015) (> 90% completeness, < 10% contamination) and assigned preliminary taxonomic classifications with Mash v2.3 (Ondov et al. 2016). NCBI plasmid database was used to identify which contigs had plasmid or genomic DNA as the origin using PlasmidFinder v.2.1.1, and NCBI AMRFinderPlus software (v3.10.5) (Feldgarden et al. 2021) was used to annotate aminoglycoside resistance genes using the NCBI AMRFinder database. AMR genes and plasmids were annotated on all contigs in the assembly. Hi-C signal was used to associated plasmids with their hosts. Thus, if a plasmid was associated with a host and had an AMR gene, the event was annotated as an AMR gene conveyed to the host via a plasmid. If an AMR gene was found on a binned contig that was not annotated as a plasmid, it was classified as an AMR gene originating from genomic DNA (Oladeinde et al. 2022). The taxonomic trees were generated with ‘’ggtree” package v3.2.1 in R (Yu 2020).

#### Plasmid transformation events detection from Hi-C data using discordant-reads analysis

Both ProxiMeta platform and bin3C v0.1.1. (Demaere and Darling 2019) tool were used for the generation of clusters to look for pBAV1K-T5-gfp integration. These available methods were not sensitive enough for detecting Hi-C links by identifying plasmid-contig (host) events. We therefore implemented a discordant reads analysis to quantify the interactions obtained from crosslinking the DNA pool between two genetic sequences that are not necessarily consecutive in the bacterial genome and accessory genome, within cells of the mixed cultures. A discordant read analysis consisted of (i) identifying which contigs from the assembly contained the GFP or Kanamycin Resistance Gene from the pBAV1K-T5-gfp plasmid, (ii) subtracting from the list containing the number of Hi-C interactions between contigs, only the contigs interacting with our plasmid genes, (iii) removing duplicated contig-contig interactions, (iv) counting the number of interactions between contig containing genes from plasmid and other microorganisms’ contigs, (v) tracing such interacting contig to which microbial cluster it belongs to assign taxonomy and (vi) normalizing the number of interactions by the length of the contig it was detected. Frequency is defined as the number of times a specific gene interacts with different contigs. The normalized number of Hi-C links is basically the frequency divided by the length (kb) of the interacting contig. This can give information about the density of the interactions. A detailed explanation of the discordant read analysis workflow and visualization of transformation events can be found in Supplementary Material Sect.  3.

## Results

### Benefit of plasmid spike for biomass growth under antibiotic treatment

**Fig. 3:**
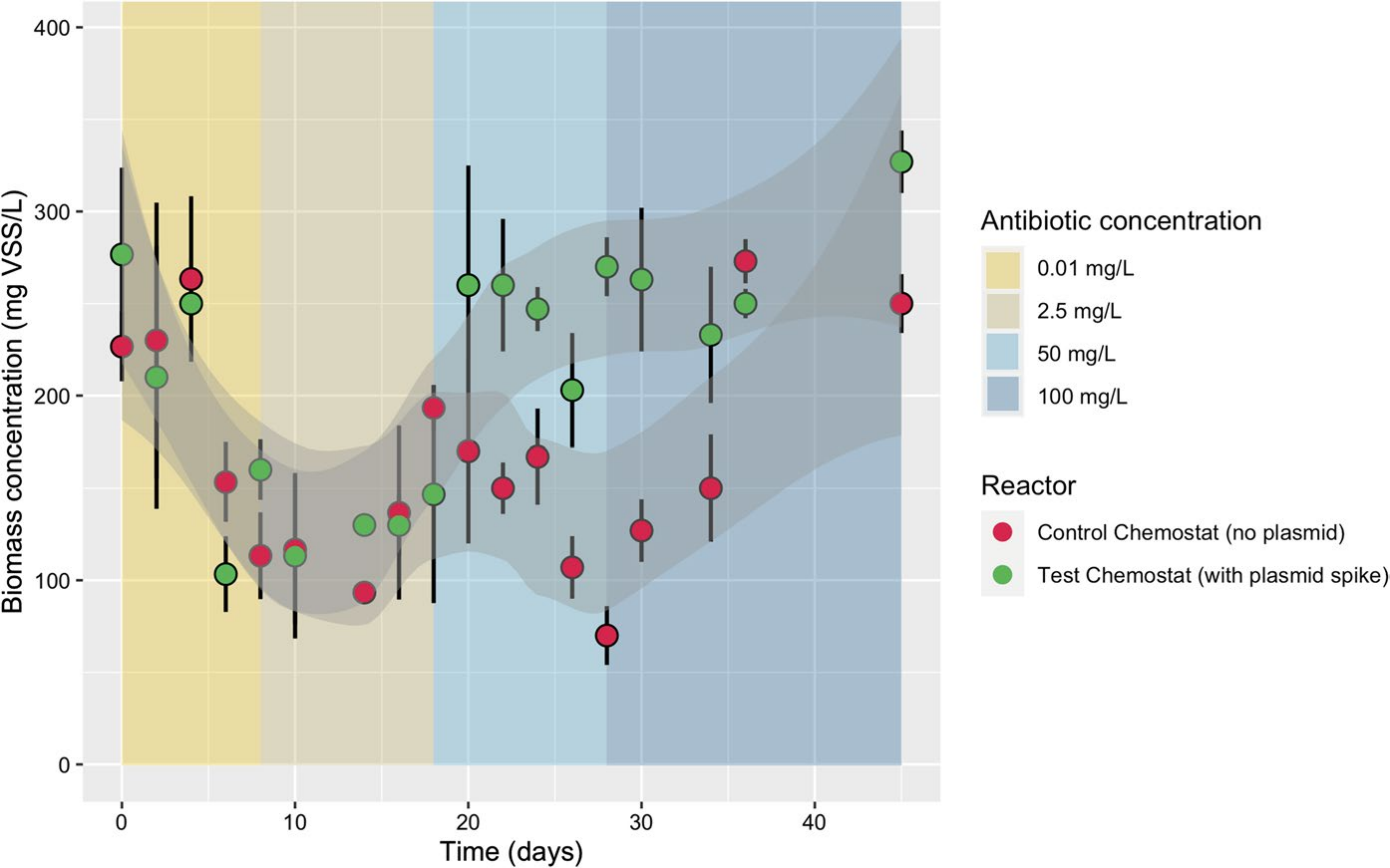
Evolution of biomass in the control and test chemostats. Daily-averaged values for volatile suspended solids (VSS) from both reactor control and reactor with free-floating plasmid over the whole operation time the experiment was conducted (45 days). Kanamycin concentrations are displayed as background-colored sections: 0.01, 2.5, 50, 100 mg L^−1^

Biomass developed in both the test and control chemostats at a growth rate (µ) of 0.042 h^−1^ equal to the imposed dilution rate, under environmental antibiotic concentrations of 0.01–2.5 mg L^−1^ of kanamycin (Fig. 3). Interestingly, under higher antibiotic pressures of 50–100 mg Kan L^−1^, a significantly higher amount of biomass accumulated in the test reactor spiked with the synthetic plasmid pBAV1K-T5-GFP that carried the kanamycin resistance (maximum difference in biomass concentration of 150 mg VSS L^−1^ when compared to the control reactor deprived of plasmid). There was significantly higher biomass at the end of all kanamycin concentration stationary phases except 2.5 mg L^−1^. When exposed to a high concentration of kanamycin of 50 mg L^−1^, the control culture displayed an abrupt decrease in viability. This control biomass was eventually able to adapt and grow under the highest antibiotic concentration of 100 mg L^−1^. Details on operation performances and regime shifts in the control and test chemostats are given in the supplementary Fig. S5.

### Microbial selection under increasing antibiotic pressure

**Fig. 4:**
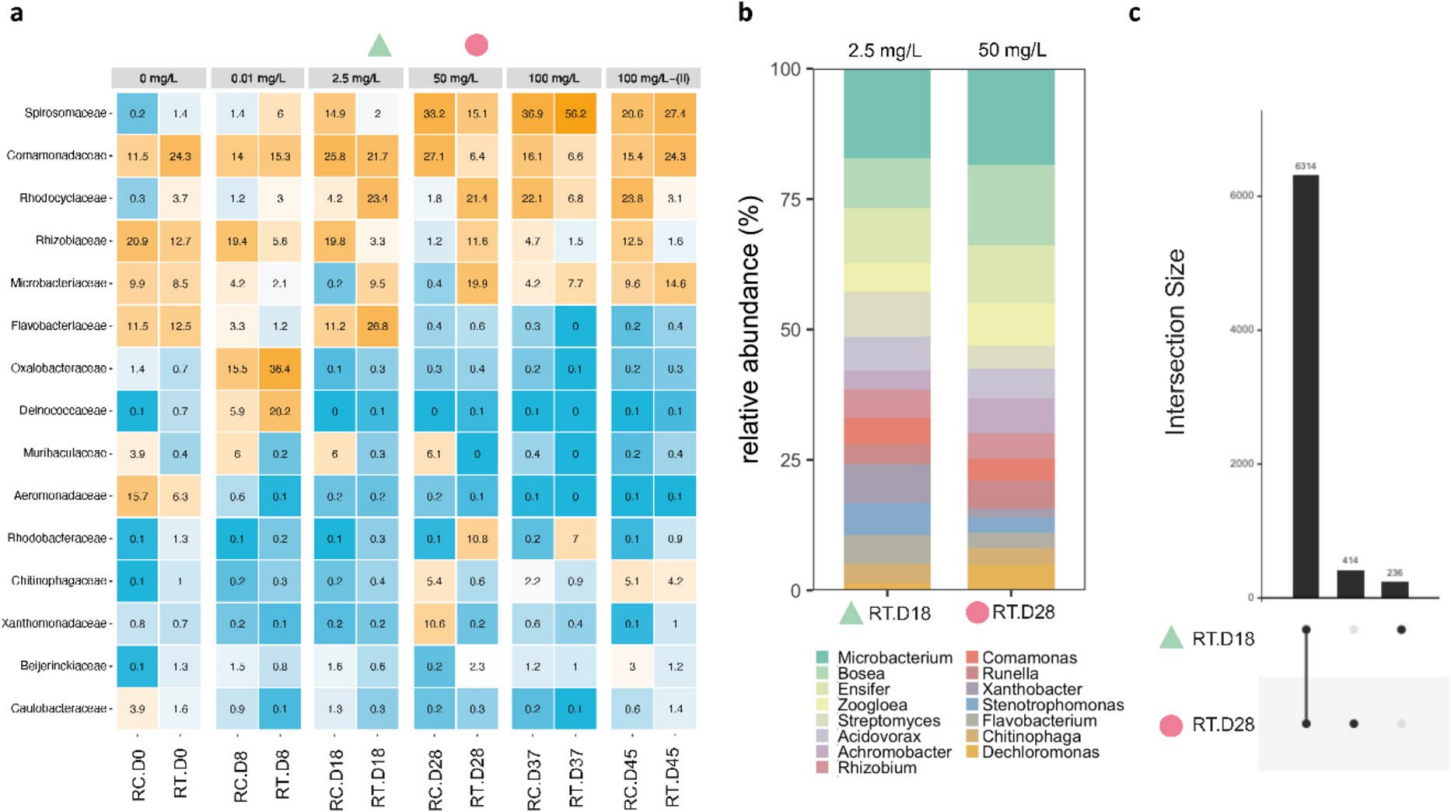
Microbial community profiles in the control and test chemostats. **a** Heatmaps from 16SrRNA amplicon sequencing showing bacterial family compositions across the operation in both chemostats grouped per kanamycin concentration (0, 0.01, 2.5, 50, 100 mg L^−1^ with spiked plasmid and 100 mg L^−1^—(II) without spiked plasmid). The different color intensities represent the relative bacterial family abundance in each population. *RC* Reactor Control without spiked plasmid. *RT* Reactor Test with spiked plasmid. (▲: Day 18–2.5 mg L^−1^) and (●: Day 28–50 mg L^−1^) represent the samples from the reactor test that were also sent to analyze for Hi-C sequencing. **b** Microbiome analysis from the samples sent for Hi-C sequencing at the genus level. **c** UpSet plot showing microbiome intersections between day 18 and day 28 from the test reactor

The compositions of the bacterial communities were first analysed by 16S rRNA gene amplicon sequencing from the mixed liquors sampled at the end of each period of antibiotic loading (0–0.01–2.5–50–100 mg Kan L^−1^) from the control (RC) and test (RT) reactors (Fig. 4a). After inoculation with activated sludge, the reactors were acclimatized for 10 days to chemostat operation prior to the start (day 0) of antibiotic supply and plasmid spikes. The same predominant bacterial populations were enriched in both reactors. Rather than the plasmid spikes, the antibiotic supply exerted the strongest selection pressure on the bacterial communities. The microbial diversity decreased with the increased antibiotic dosage. Five families of *Spirosomaceae, Comamonadaceae, Rhodocyclaceae, Microbacteriaceae,* and *Chitinophagaceae* were enriched under kanamycin exposure.

The metagenomes of biomasses collected at 2.5 and 50 mg Kan L^−1^ shared 91% of sequencing reads that mainly affiliated with *Microbacterium, Bosea, Ensifer, Zoogloea,* and *Streptomyces* genera (Fig. 4b, c). Genome clusters were recovered for these populations by aligning Hi-C reads against the shotgun metagenomic de novo assembly, from both timepoints (supplementary Figs. S3 and S4).

### Accumulation of the spiked plasmid in the microbial system at high antibiotic exposure

**Fig. 5:**
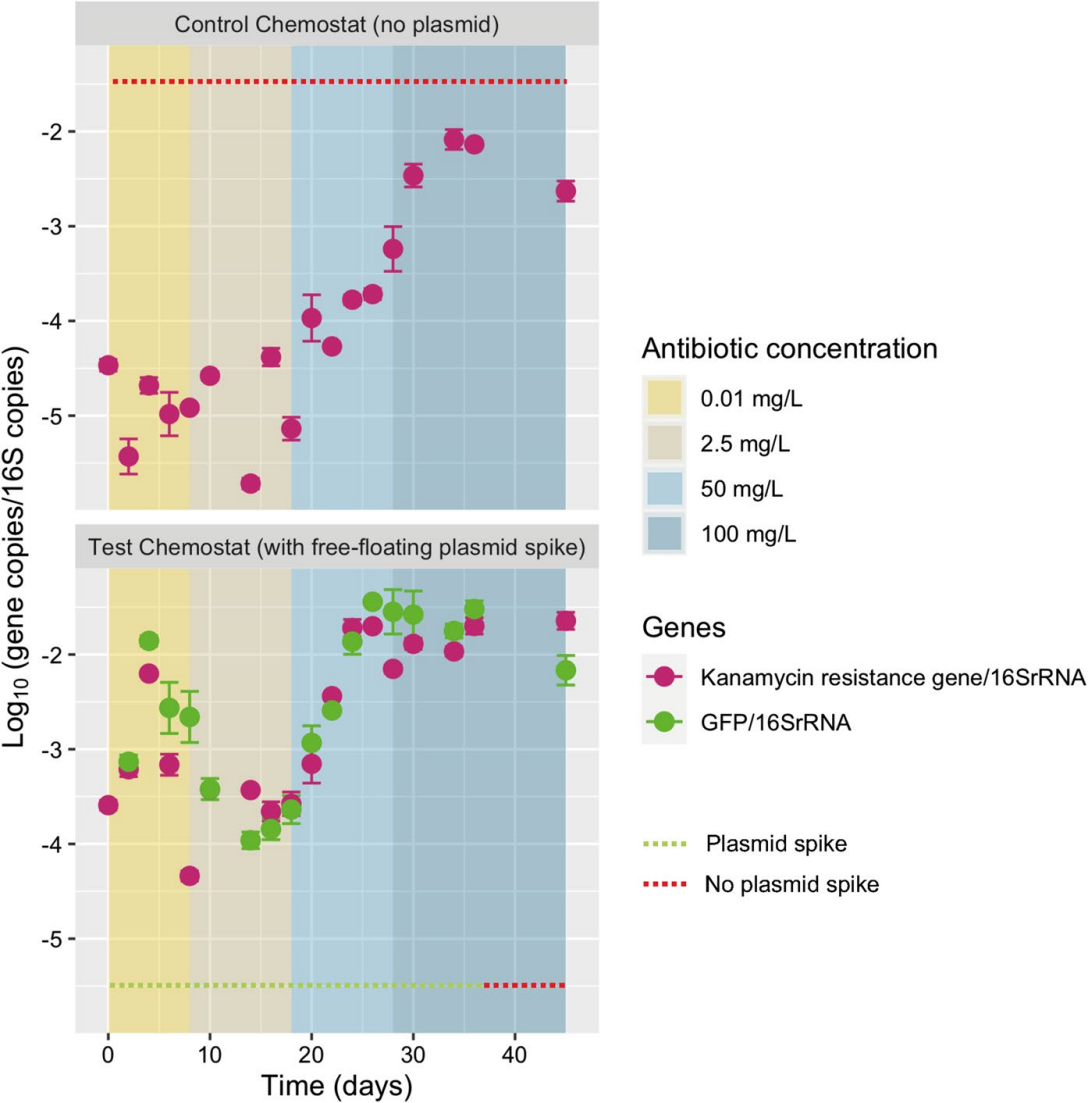
Relative abundance of GFP and kanamycin resistance genes detected from the plasmid pBAV1K-T5-GFP relative to 16S rRNA gene in every sampling point. Only data from the reactor with free-floating plasmid displayed as non-detected values were detected for reactor control. Kanamycin concentrations are displayed on colored-background sections: 0.01, 2.5, 50, 100 mg L^−1^. The plasmid was not spiked anymore in the test reactor (after day 36)

The abundance of the *kanR* and GFP genes (that were carried by the spiked plasmid) was quantified by quantitative PCR (qPCR) throughout the operation of both the control reactor (no plasmid spike) and the test reactor (plasmid spike) (Fig. 5, upper and lower panels, respectively).

A kanamycin-resistant enrichment culture developed in both reactors alongside the increasing antibiotic dosage. The *kanR* gene was obtained by the microorganisms either exogenously by transformation from the spiked plasmid or endogenously from the microbes present in the activated sludge inoculum or both.

The GFP gene was only detected in the test reactor that was spiked with the plasmid, but not in the control reactor. The ratios of the abundances of both the GFP and *kanR* genes over the abundance of the bacterial *16S rRNA* gene increased in the biomass of the test reactor at high antibiotic concentration. The ratio of GFP/16S rRNA gene copies rose in the test mixed culture from -3.5 to -1.5 log gene copies. This indicated that the spiked plasmid accumulated in the microbial system, in addition to the enrichment of kanamycin-resistant populations.

After an exposure of 18 days to the highest kanamycin concentration of 100 mg L^−1^, the test culture was maintained for an additional week during which no plasmid was spiked. The resulting GFP/16S rRNA gene ratio did not decrease, supporting the hypothesis that the plasmid was integrated into microbial populations selected in the mixed culture.

Hence, Hi-C sequencing was then used to uncover transformation events and to identify the microbial transformants in the microbial community.

### Microbial transformants detected in the mixed culture by Hi-C sequencing

Capturing the transformation events in a microbial community spiked with a specific plasmid is a challenge. Hi-C sequencing enables the detection of microorganisms that effectively carry plamisds, by ligating them to the genomic DNA prior to extracting the full DNA pool and sequencing it by metagenomics.

**Fig. 6:**
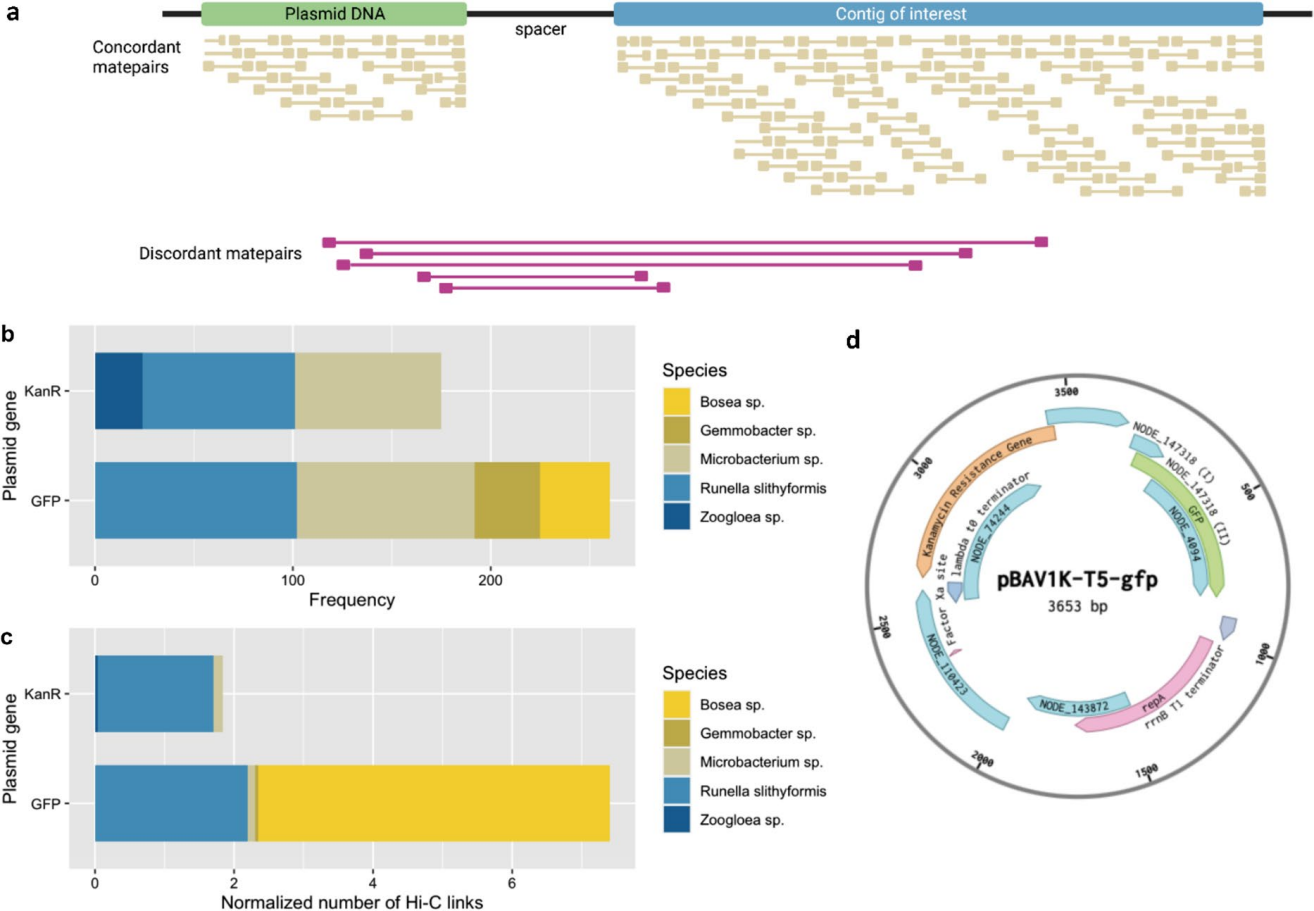
Detection of microorganisms transformed by the synthetic plasmid using Hi-C sequencing. **a** Schematic representation of the discordant read analysis performed on contigs interacting with plasmid contigs, where synthetic constructs were generated to quantify the number of interactions from the Hi-C library alignment. **b**, **c** Taxonomic assignment of contigs that were linked to contigs harboring the information of the spiked pBAV1K-T5-GFP plasmid by frequency and by a normalized number of Hi-C links (#events divided by the length of the interacting contig). **d** Plasmid map containing the contigs used for the discordant read analysis. The *kanR* information in **a**, **b** is a combination of the number of interactions between the Kanamycin Resistance Gene with the NODE_74244 and NODE_147318 (I). Likewise, the GFP information is a combination of the number of interactions between the *GFP* gene with the NODE_147318 (II) and NODE_4094

From the Hi-C metagenomes sequenced at low (2.5 mg Kan L^−1^, RT.D18) and high (50 mg Kan L^−1^, RT.D28) antibiotic pressures, synthetic constructs consisting of the plasmid sequence, the non-coding spacer DNA, and the metagenome contigs displaying high Hi-C links to plasmid contigs were generated. The transformation of bacterial populations with the spiked plasmid was verified, together with its integration into their genome or its episomal presence in their cytoplasm (Fig. 6a and Supplementary Figs. S6, S7). Contigs interacting with the *kanR* or GFP gene sequences of the synthetic plasmid were used to quantify the number of Hi-C links (Fig. 6d).

No synthetic plasmid sequence was retrieved from the raw reads and from the assembly of the RT.D18 biomass sampled at low kanamycin concentration. At this time point, the plasmid was either present at a very low concentration (which corresponds to the lowest plasmid/16S ratio detected by qPCR; Fig. 5), but metagenomics was not sensitive enough to detect it.

Conversely, the assembly of the RT.D28 biomass exposed to a high kanamycin loading harboured multiple contigs containing genetic information from the spiked plasmid. Activated sludge microorganisms were therefore able to acquire the exogenous synthetic plasmid through transformation. The main microbial transformants affiliated with the Gram-negative *Bosea*, *Runella, Gemmobacter* and *Zoogloea* spp., and with the Gram-positive *Microbacterium* sp.. Their genomic DNAs were cross-linked with genetic fragments originating from the spiked plasmid (Fig. 6b). Contigs of *Runella slythiformis* displayed the highest frequency of Hi-C links (102), followed by *Microbacterium oxydans* (90) (Fig. 6b). *Bosea* sp. displayed the highest number of Hi-C links per kb of contig sequence length (5.1 Hi-C links kb^−1^), followed by *Runella* spp. (2.2 Hi-C links kb^−1^) (Fig. 6c). Cross-links with the full plasmid were visualised to demonstrate the plasmid presence in the microbial cells: this significantly demonstrated that *Bosea* and *Runella* spp*.* acquired the synthetic plasmid in the microbial community (Supplementary Figs. S8–S11).

The relative abundance of these competent populations increased by 2–4 times in the microbiome when switching the kanamycin concentration from 2.5 up to 50 mg Kan L^−1^. In biomass concentration equivalence terms, they were able to grow under this higher antibiotic pressure: *Microbacterium* from 5.4 to 14.6 mg VSS L^−1^, *Bosea* from 3 to 12.4 mg VSS L^−1^, *Runella* from 1.6 3.3 mg L^−1^; and *Zoogloea* from 1.74 to 6.6 mg VSS L^−1^. These microorganisms belong to distant classes, namely *Alphaproteobacteria, Actinobacteria, Bacteroidetes* and *Betaproteobacteria*. The Hi-C analysis thus informed that phylogenetically distant microorganisms were transformed with the synthetic plasmid in the mixed culture (Supplementary Fig. S12). Transformation was detected when the microbial system was exposed to a sufficient selection pressure, here, a high antibiotic loading.

### Detection of natural plasmids carrying aminoglycoside resistance genes

**Fig. 7:**
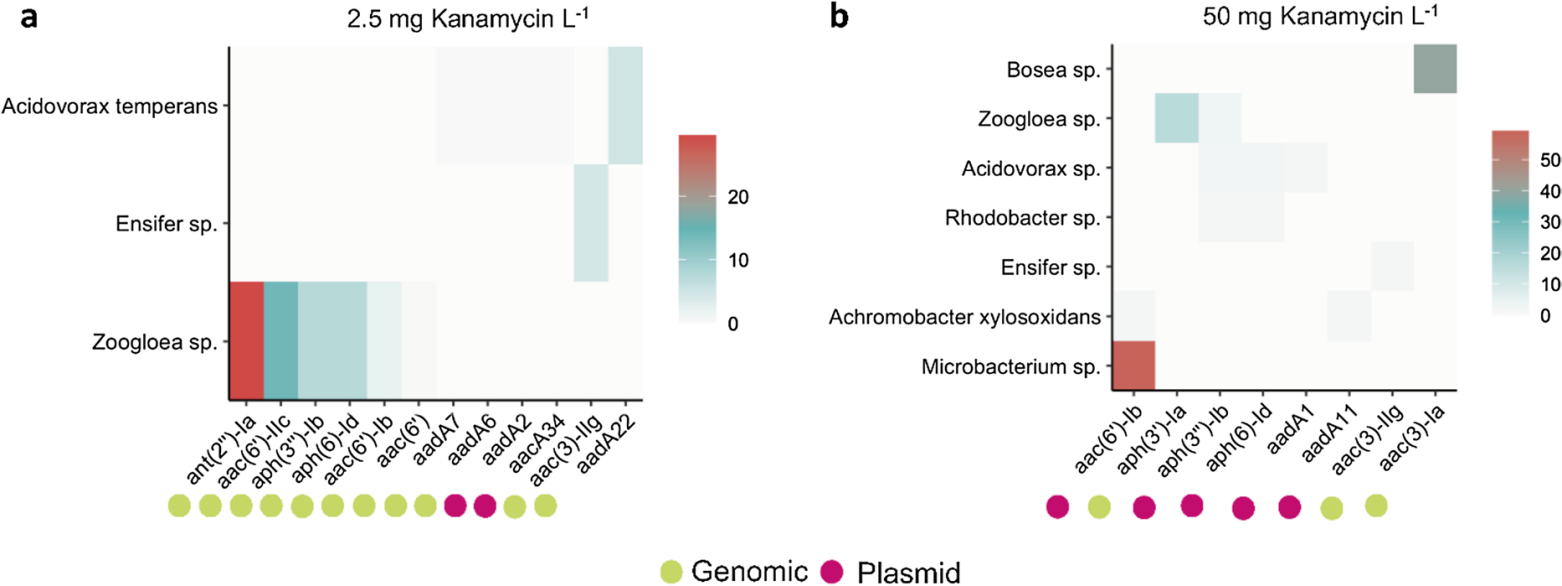
Detection of plasmids carrying aminoglycoside resistance in the microbial community by Hi-C sequencing. Comparison of the number of Hi-C interactions normalized by contig length (kb) between aminoglycoside resistance genes and bacterial hosts under exposures to kanamycin concentrations of 2.5 (**a**) and 50 (**b**) mg L^−1^ in the test chemostat. Contigs containing aminoglycoside resistance genes were classified to determine their origin: genomic or plasmid DNA. Colors represent the number of Hi-C interactions normalized by the contig length (kb)

Increasing concentrations of the aminoglycoside antibiotic kanamycin did not only stimulate the uptake of the synthetic plasmid carrying the *kanR* and GFP genes, but also induced a larger presence of a diversity of aminoglycoside resistance genes in the bacterial populations resistant to kanamycin and selected in the mixed culture (Fig. 7). Hi-C sequencing enabled to unlock the mobilisation of aminoglycoside resistance genes as well.

In the Hi-C sequenced test culture, the increase in kanamycin from 2.5 to 50 mg L^−1^ doubled the number of bacterial populations (from 3 to 7, respectively) with genomic sequences interacting with aminoglycoside resistance genes. The maximum number of Hi-C links between aminoglycoside resistance genes and genomic sequences of bacterial hosts also doubled.

At 2.5 mg Kan L^−1^, the aminoglycoside resistance genes were mainly detected in the genomic DNA sequences. Intra-genome Hi-C links within the chromosome were detected within three microbial populations (*Acidovorax temperans*, *Ensifer* sp., *Zoogloea* sp.). *Zoogloea* sp. contained multiple aminoglycoside resistance genes linked within sequences of its genome, such as 29 Hi-C links kb^−1^ with the *ant(2’’)-Ia* gene (Fig. 7a) that codes for the aminoglycoside-2″-O-nucleotidyltransferase that is a major determinant of enzyme-dependent resistance to aminoglycosides (Hirsch et al. 2014).

At 50 mg Kan L^−1^, aminoglycoside resistance genes were mainly detected on the accessory genomes of the microbial populations, *i.e.*, on natural plasmids that were present in the cells. We detected multiple aminoglycoside resistance genes encoded in plasmids (Fig. 7b). For instance, *Microbacterium* sp. displayed as high as 59 Hi-C links kb^−1^ between the aminoglycoside N-acetyltransferase gene *aac(6’)-Ib* present on natural plasmids and its genomic DNA sequence. The genomes of four additional populations (*Achromobacter xylosoxidans*, *Rhodobacter* sp., *Acidovorax* sp., *Zoogloea* sp.) were cross-linked with aminoglycoside resistance genes present on natural plasmids, while intra-genome Hi-C links were also abundant in *Bosea* sp. and *Zoogloea* sp.. Some resistance genes carried by plasmids were found in multiple bacteria, such as the aminoglycoside 3’-phosphotransferase gene *aph(3’’)-Ib* in *Zoogloea, Acidovorax* and *Rhodobacter* spp..

Antibiotic pressure therefore resulted not only in the uptake of the spiked synthetic plasmid carrying *kanR* genes, but also in the proliferation of bacterial hosts that contained more copies of genes coding for aminoglycoside hydrolyzing enzymes either on their genomic DNA or on naturally occurring plasmids. Interestingly, a shift in the origin of these aminoglycoside resistance genes was detected from genomic DNA to plasmid DNA with the increase in antibiotic loading, indicating the mobilisation of aminoglycoside resistance by the microbial populations within the community exposed to kanamycin.

## Discussion

### Environmental microorganisms can get transformed with an exogenous synthetic plasmid

Our study highlights that microorganisms originating from an activated sludge environment of a wastewater treatment plant can get transformed with an exogenous synthetic plasmid. Transformation was the only possible transfer mechanism since this rolling-circle plasmid was deprived from any conjugative property.

Transformation was mainly detected in Gram-negative bacteria like the *Bosea*, *Runella*, *Gemmobacter* and the well-known activated sludge floc former *Zoogloea* spp. (Weissbrodt et al. 2012), as well as the Gram-positive *Microbacterium* sp. (Fig. 6). The plasmid was acquired by these taxonomically distant populations affiliating with classes as wide as *Alphaproteobacteria, Betaproteobacteria, Bacteroidetes*, and *Actinobacteria*, that are known to contain one or more naturally competent species (Mell and Redfield 2014).

From metagenomic analyses of a full-scale WWTP, we recently showed that most microorganisms that harboured ARGs that co-localized with MGEs on DNA contigs were Gram-negative (Calderón-Franco et al. 2022). These bacteria possess an outer membrane which protect them against antibiotics, making them more difficult to suppress by antimicrobial treatment. On the other hand, Gram-positive bacteria harbour a thick outer layer of peptidoglycans that absorbs antibiotics and detergents easier, leading to a faster cell death and a slower development of resistance (Jubeh et al. 2020).

High kanamycin pressure stimulated the accumulation of the synthetic plasmid carrying resistance among the wastewater microorganisms (Fig. 5). In addition, the antibiotic treatment resulted in a higher natural occurrence of aminoglycoside resistance genes located on plasmids hosted by bacterial populations of the biomass (Fig. 7). Hence, the populations selected under antibiotic exposure comprised both the microorganisms that took up the synthetic plasmid as well as other non-transformed bacteria which developed a natural resistance to the antibiotic. Using Hi-C sequencing, we highlighted these two mechanisms for the mobilisation of resistance genes under antibiotic pressure. When bacteria are exposed to high concentrations of antibiotics, they may undergo selection pressure, leading to the enrichment of resistant strains. This selective pressure can also result in the transfer of ARGs from the chromosome to plasmids, or the acquisition of new plasmids carrying ARGs.

Zhao et al. (2020) already showed how antibiotic selection pressures significantly increased the abundance and proportions of ARGs mediated by plasmids (57.9%) in wastewater environments, being more prevalent than those encoded in chromosomes (19.2%). Moreover, higher co-occurrence frequency of ARGs and MGEs revealed that antibiotics enhanced the mobility potential of ARGs mediated by both plasmids and integrative and conjugtive elements.

### Accumulation of the exogenous plasmid promoted growth at high antibiotic exposure

The biomasses from both the test and control reactors were able to adapt and to grow substantially under an antibiotic loading as high as 100 mg Kan L^−1^ (Fig. 5). In these replicated reactors, the test biomass, that was spiked with the plasmid containing the *kanR* and GFP genes and accumulating in the microbial system, developed faster already at 50 mg Kan L^−1^ and yielded a higher production. The *kanR* gene carried by the synthetic plasmid conferred resistance against the antibiotic, allowing the microbial transformants to improve their fitness, withstand, and grow under acute antibiotic exposure.

The qPCR results nonetheless showed that microorganisms that harboured *kanR* genes were already present in the activated sludge inoculum collected at the full-scale WWTP. This is not surprising since genes coding for resistances to aminoglycosides have been identified in the influent, activated sludge, and effluent of WWTPs (Petrovich et al. 2018; Pärnänen et al. 2019; Yoo et al. 2020). However, by tracing both the *kanR* and GFP gene carried by the synthetic plasmid, we could see that bacterial growth resulted from plasmid presence in the system. qPCR analysis of the *GFP* gene showed that the plasmid remained in the test chemostat even 8 days (i.e., after 8 hydraulic and mean cell retention times, or 12 number of generations) after we halted the plasmid spikes. The plasmid remained either transformed inside the selected microorganisms or adsorbed to extracellular polymeric substances (Das et al. 2013). Proximity-ligation Hi-C sequencing allowed to detect the effective transformation of the microorganisms by quantifying the links between the intracellular pools of genomic DNA and the plasmid DNA, within the microbial community.

### Transformation can disseminate resistance determinants among microbial communities

Antibiotics are typically detected in concentrations spanning from 0.01 µg L^−1^ in the sea to 0.1 µg L^−1^ in rivers, 1 µg L^−1^ in treated municipal sewage, 10 µg L^−1^ in untreated sewage, up to as high as 0.1–10 mg L^−1^ in untreated hospital effluents and industrially polluted surface water, and above 100 mg L^−1^ in the gut during medical administration of antibiotics (Larsson and Flach 2022). In this work, transformation was detected under high antibiotic exposure (> 50 mg L^−1^) resembling the latter polluted and gut environments loaded by antibiotics. We used high antibiotic levels to detect a clear response from the microbial system. Here, transformation was mainly promoted under a substantial environmental pressure like high antibiotic concentration. Even though synthetic plasmids are less likely to reach the microbial gut, our results highlight that transformation can be an important mechanism of AMR transfer in a microbiota exposed to high antibiotic concentrations like in the gut (Blokesch 2016).

Catching movements of one single plasmid type in the metagenome of a complex microbial community is challenging. Tracking the temporal dynamics of plasmids uptake via transformation and other HGT processes such as conjugation or transduction in the most recurrent microbial hosts and spreaders in communities is an important fundamental aspect. This will also become an important step toward assessing risks associated with the dissemination of xenogenetic elements that comprise ARGs, MGEs as well as genetically modified biosynthetic gene clusters. The consequences of single transformation events can be vast (Larsson and Flach 2022), such as the development of “superbugs” resistant to two or more antibiotics that can induce severe medical issues, notably if multi-resistant pathogenic bacteria enter the drinking water and food chain. To increase the detection of transformation events, the resolution of Hi-C metagenomic sequencing can be combined with the high sensitivity of epicPCR (emulsion, paired isolation, and concatenation PCR) (Spencer et al. 2016). This can help identify target functional genes fused to 16S rRNA genes in amplicons produced from the DNA pool of competent microbial populations. Future research should also uncover the environmental conditions (*e.g.*, nutrient limitations, temperature shifts) and genetic traits of the exDNA material (*e.g.*, linear or plasmid DNA, methylation patterns) that trigger microbial competence, exDNA uptake, and genetic exchange within microbiomes like activated sludge.

In conclusion, our work shows that activated sludge microorganisms can get transformed by a synthetic plasmid present in their wastewater environment. Microbial transformants were selected in the community at a high antibiotic pressure. In addition to the uptake of this exogenous plasmid, the exposure to kanamycin resulted in the accumulation of aminoglycoside resistance genes on the natural plasmidome of bacterial populations selected in the mixed culture. This led to the development of biomasses able to resist antibiotic concentrations as high as prevailing in heavily polluted environments or under antibiotic ingestion in the gut. The main hosts of the synthetic and naturally occurring plasmids carrying resistance can lead to infections (*e.g.*, *Microbacterium*, Bosea), when humans and animals are exposed to biologically contaminated water resources. The participation of the floc-forming *Zoogloea* sp. in the mobilisation process of resistance plasmids shows that native activated sludge microorganisms that contribute to nutrient cycles can also participate in the dissemination of xenogenetic elements. Transformation requires a specific consideration in the surveillance of AMR propagation across the water and health chain. From a risk perspective, the acquisition of synthetic DNA materials by microorganisms in urban and natural environments needs to be prevented. Beyond the wastewater environment, transformation contributes to the emergence and proliferation of antibiotic-resistant pathogens in human microbiota and the spread of virulence factors (Blokesch 2016, 2017). Our approach combining mixed-culture biotechnology and Hi-C metagenome sequencing uncovered the transformability of microorganisms in a microbial community impacted by antibiotics. Further combination to transcriptomic and proteomic research on the expression of competence genes and proteins can help elucidate the mechanisms and phenotypes by which the bacterial transformants acquire and re-use the free DNA materials as a selective and evolutionary advantage in a microbiome.

## Conclusions

We identified that microorganisms in a mixed culture enriched from activated sludge could take up and get transformed by synthetic plasmids present in their wastewater environment, provided a selection pressure is present, like a high antibiotic concentration. The result of this work involving mixed-culture biotechnology and Hi-C sequencing led to the following additional main conclusions:

1. The spike of plasmid DNA helped the biomass adapt and to have a higher yield under high antibiotic concentrations (> 50 mg L^−1^).
2. The plasmid DNA accumulated in the test chemostat even when its spiking was stopped for 8 days, by either uptake inside bacterial cells and/or adsorbed to the EPS.
3. High kanamycin concentrations (50–100 mg L^−1^) promoted the mobility of aminoglycoside resistance genes among bacterial hosts.
4. Environmental antibiotic concentrations (≤ 2.5 mg L^−1^) did not induce detectable transformation events, while lab-selection concentrations (50 mg L^−1^) did.
5. The main hosts containing the spiked synthetic plasmid pBAV1K-T5-GFP in the activated sludge were the Gram-negative bacteria *Bosea* sp. and *Runella* sp. (accompanied by *Gemmobacter* sp. and the well-known activated sludge floc former *Zoogloea* sp), and the Gram-positive *Microbacterium* sp..

## Supplementary Information

Below is the link to the electronic supplementary material.

Supplementary file1 (DOCX 15128 KB)

## Data Availability

Metagenome sequencing and amplicon sequencing data were deposited in the NCBI database with the BioProject ID: PRJNA868937.
